# Poly[μ_2_-chlorido-nona­methyl-μ_3_-nitrato-tritin(IV)]. Corrigendum

**DOI:** 10.1107/S1600536808017091

**Published:** 2008-06-21

**Authors:** Sadiq-ur-Rehman, Saira Sherzaman, Saqib Ali, Saira Shahzadi, Madeleine Helliwell

**Affiliations:** a Department of Chemistry, University of Azad Jammu and Kashmir, Muzaffarabad, Pakistan; b Department of Chemistry, Quaid-i-Azam University, Islamabad 45320, Pakistan; c Department of Chemistry, G.C. University, Faisalabad, Pakistan; d School of Chemistry, The University of Manchester, Manchester M13 9PL, England

## Abstract

Corrigendum to *Acta Cryst.* (2007), E**63**, m2329.

An error in the original formulation of the title compound in the paper by Sadiq-ur-Rehman, Sherzaman, Ali, Shahzadi & Helliwell [*Acta Cryst.* (2007), E**63**, m2329] is corrected.The title compound in the paper by Sadiq-ur-Rehman, Sherzaman, Ali, Shahzadi & Helliwell [*Acta Cryst.* (2007), E**63**, m2329] was an unexpected product which seemed to have nitrate coordinated to three Sn atoms. However, it was noticed that the charges do not balance and that it is most likely that the nitrate is in fact a carbonate. Regrettably, there is no material to carry out microanalysis, but a plausible mechanism has been suggested to explain the unexpected formation of the product. Trimethyl­tin chloride will react with methanol in the presence of a base (4-hydroxy­piperidine) to give trimethyl­tin methoxide, which will rapidly hydrolyze in air to give the hydroxide. Both the methoxide and the hydroxide will react with atmospheric CO_2_ to give the carbonate (Bloodworth *et al.*, 1967 ▸; Blunden *et al.*, 1984 ▸; Sato, 1967 ▸). Me_3_SnCl + MeOH + base → Me_3_SnOMe + base·HCl Me_3_SnOMe + H_2_O → Me_3_SnOH + MeOH Me_3_SnOH + CO_2_ → Me_3_SnOCO_2_HMe_3_SnOCO_2_H + Me_3_SnOMe → Me_3_SnOCO_2_SnMe_3_ + MeOH. The carbonate then forms a coordination copolymer with trimethyl­tin chloride. The name of the title compound is corrected to poly[μ_3_-carbonato-μ_3_-chlorido-nona­methyl­tri­tin(IV)], [Sn_3_(CH_3_)_9_(CO_3_)Cl] (*M* *_r_* = 586.84).
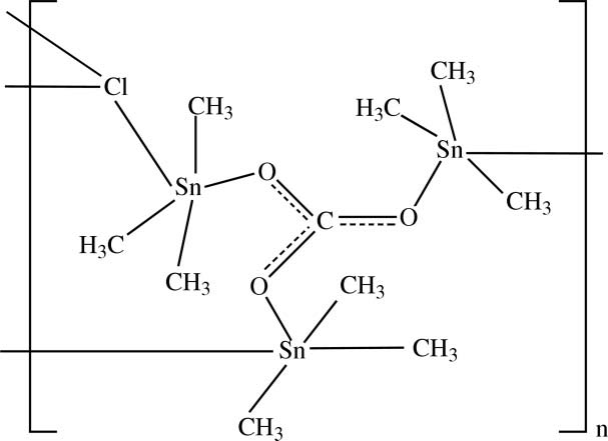


## Supplementary Material

Crystal structure: contains datablocks global, I. DOI: 10.1107/S1600536808017091/ez9088sup1.cif

[Chem scheme1]. DOI: 10.1107/S1600536808017091/ez9088fig1.tif
The structure of (I), showing part of a polymeric sheet with atoms of the asymmetric unit labelled. H atoms have been omitted for clarity.
