# Supplementary figures and images for: Poly[μ2-chlorido-nona­methyl-μ3-nitrato-tritin(IV)]. Corrigendum
Source: Acta Crystallogr Sect E Struct Rep Online. 2008 Jun 21;64(Pt 7):e26. doi: 10.1107/S1600536808017091 (PMC2961754; doi:10.1107/S1600536808017091)

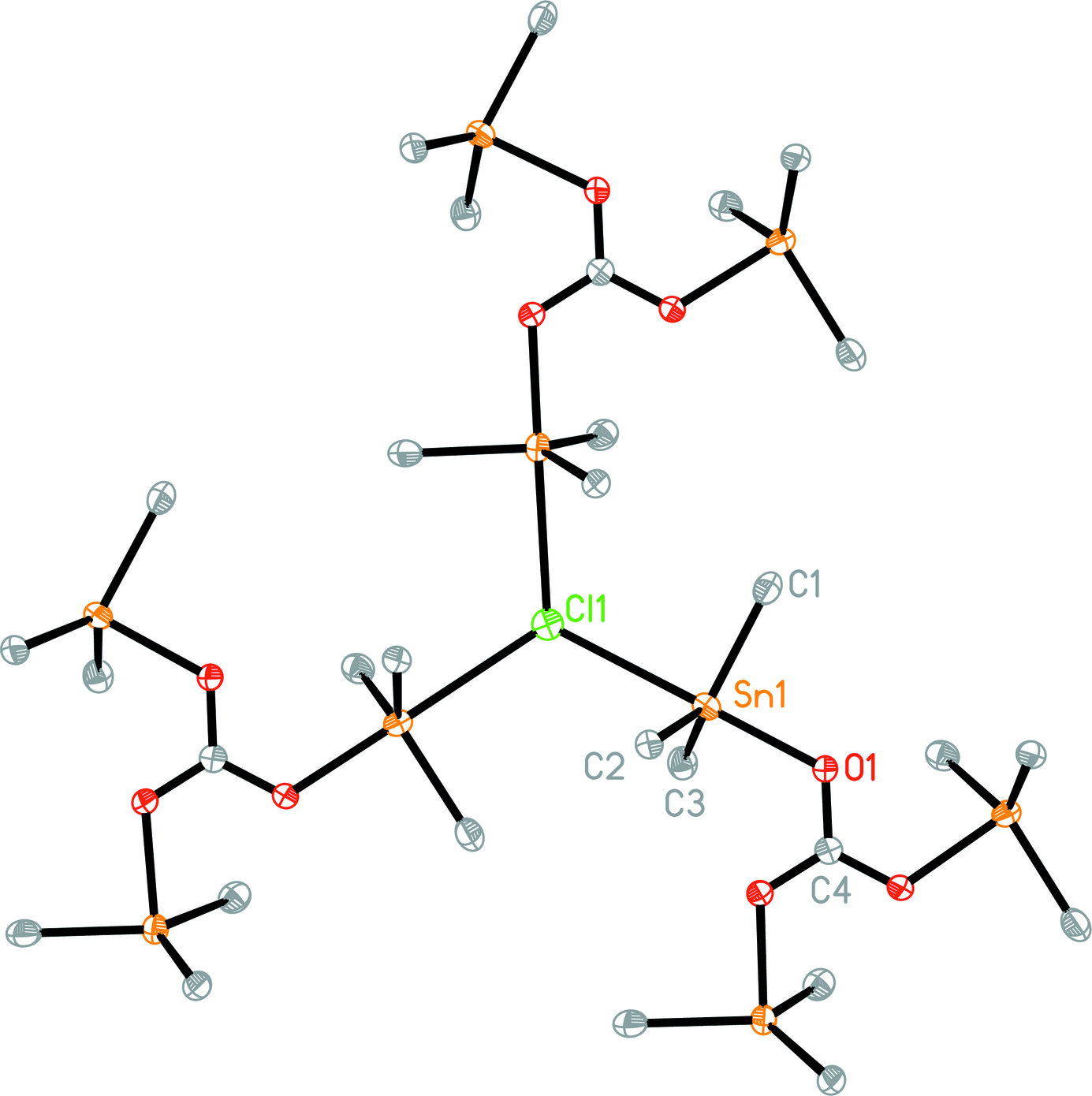

Supplement: Supplementary file 2 [file e-64-00e26-fig1.tif]
